# Challenges in hybrid management in healthcare: a study of the interplay between divisional managers and clinical directors in a decentralized healthcare organization in Sweden

**DOI:** 10.1186/s12913-025-13977-y

**Published:** 2026-01-12

**Authors:** Mikael Ohrling

**Affiliations:** 1 Department of Learning, Informatics, Management and Ethics, Medical Management Centre, Karolinska Institutet, Stockholm, Sweden; 2 Stockholm Health Care Services, Region Stockholm, Sweden; 3 Institutionen för lärande, informatik, management och etik, Karolinska Institutet, Stockholm, 171 77 Sweden

**Keywords:** Hybrid management, Clinical directorates, Decentralization, Healthcare governance, Organizational change

## Abstract

**Background:**

The integration of medical professionals into management—so-called hybrid management—has emerged as a key strategy in public healthcare reforms. Clinical directorates (CDs) are often seen as vehicles for this integration. While prior studies have explored hybrid management roles and decentralization, less is known about how multiple hybrid-managerial levels interact within one organization. This study examines the perceptions of clinical directors in a large, decentralized healthcare provider in Sweden, following the introduction of a divisional hybrid-managerial level intended to enhance efficiency and coordination.

**Methods:**

A sequential explanatory mixed-methods approach was used, combining two web-based surveys (2018 and 2019) sent to all clinical directors (*n* = 95). The responses were analyzed quantitatively via nonparametric tests (Mann‒Whitney exact test, Fisher’s exact test) and indices (polarity, intensity, uncertainty). The open-ended responses were analyzed via directed content analysis to deepen the interpretation of the quantitative trends.

**Results:**

Response rates were high (84% in 2018; 97% in 2019). The introduction of a divisional hybrid-managerial level yielded mixed results. Some improvements in coordination and leadership support, particularly in mental health care services, have been reported. However, many clinical directors—especially in primary care—perceived increased administrative burden, unclear roles, and confusion over responsibilities. Statistical analysis confirmed significant changes in perceived time for patient care and administrative burden but not in central support. The qualitative data revealed that while some directors appreciated improved strategic dialog, others cited increased complexity and ineffective support systems.

**Conclusions:**

Introducing a divisional hybrid-managerial level in a decentralized healthcare setting can improve coordination but risks adding bureaucratic complexity without clear role definitions and adequate support. The study underscores the importance of clarifying authority and responsibilities when layering hybrid roles. Effective hybrid management requires more than professional alignment—it demands robust systems, clear communication, and a culture that reconciles managerial and professional logics. These findings offer insights for health systems pursuing hybrid governance models and contribute to theory on decentralized management in complex service organizations.

**Supplementary Information:**

The online version contains supplementary material available at 10.1186/s12913-025-13977-y.

## Introduction

The incorporation of medical professionals into management is a multifaceted strategy [1–4]. Early reforms shifted from a command-and-control model with administrative budget holders separated from clinicians into new organizational forms [5]. New public management (NPM) principles increased the demand for managerial knowledge in clinical operations, especially for contracting and performance management [2, 6]. This shift from dual management resulted in hybrid management roles, where clinicians became involved in a new way [7]. After the fallacy of the NPM reforms, hybrid roles were considered more to integrate managerial and professional dimensions for improvement than just control [8–10].

Clinical directorates (CDs) are a common way to involve medical professionals in management [5, 10], and the model has expanded to support performance and service improvement [3, 11, 12]. CDs are mainly studied in hospital settings [2, 5, 13] but also in primary and community care [12, 14]. Research shows that doctor-managed hospitals perform better [15, 16], and medical professionals on board are positively associated with quality improvement [3, 17]. CDs can, however, exist at multiple organizational levels [5], creating a hierarchal chain of hybrid managerial roles.

Despite the substantial literature on improving healthcare services by introducing hybrid management and CDs, there is a knowledge gap around the interplay between hybrid-managerial levels within an organization. Correia and Denis [13, p 74] stress the importance *of understanding the “microlevel processes of role configuration and relations among hybrids and their medical peers*”.

This study addresses that gap by examining how clinical directors in a large, decentralized healthcare organization in Sweden perceived the introduction of a new divisional hybrid-managerial level **i**ntended to improve efficiency and coordination. Building on previous research on the same organizational model [12, 14], this article explores the interplay between hybrid roles across managerial levels and their implications for healthcare governance and decentralization.

This article is structured as follows. First, the setting and the theoretical frameworks will be presented to understand hybrid management in relation to organizational structures and processes in the study setting. Then, empirical data from two surveys are used to illustrate how the introduction of a hybrid-managerial divisional level to increase efficiency is perceived by clinical directors. Finally, these findings will be discussed in relation to clinical directorates, hybrid management, and decentralization.

### Study setting

The regional public health provider in Stockholm, Sweden, manages its hospitals and services as separate entities, but in 2004, all public primary, community and mental healthcare services were merged into one organization serving 2.4 million inhabitants The organization consists of 122 decentralized CDs divided into 700 centers or units, Table 1 [18]. In the mixed public-private healthcare system, private providers are contracted by the regional Commissioner but governed by their owners [19], while public services have a professional board appointed by the regional general assembly and are likewise contracted by the Commissioner.

**Table 1:** Facts on the healthcare services organization

Services	PHC	MHCS	GASE	HA	RD*	Total
Revenue 2018 (million Euro)	320	480	130	150	32	1 112
No of employees	3 600	5 500	1 050	960	360	11 470
No of outpatient visits	3 776 272	1 208 931	502 810	157 108	-	5 645 121
No of beds	-	875	246	-	-	1 21
No of clinical directorates	95	9	7	6	5	122
No of clinical directors	68	9	7	6	5	95

PHC = Primary healthcare; MHCS = Mental healthcare services; GASE = Geriatrics, Advanced palliative home care, Somatic specialist care, Emergency centers; HA = Habilitation and assistive technology; RD = Research and development; *) from 1 Oct 2017

The organization is a highly decentralized line-managed public professional bureaucracy with value- and trust-based governance with about 650 managers [12]. This offers a unique setting for studying interactions between managerial levels when a divisional level is introduced into a CD-based decentralized structure.

After a decade of broad autonomy for clinical directors, a need for stronger coordination, boundary-spanning and reduced local administration led to the introduction of a new divisional management level in 2016, while clinical directors retained their authority. Divisional hybrid managers—former clinical directors—were appointed tasked with appointing and coordinating clinical directors to improve consolidation and efficiency, and a fifth R&D division was added in 2017. The reform aimed to free up clinical time, reduce administrative burden and strengthen administrative competence.

### Study objectives

To address the knowledge gaps, the objectives of this study were to (1) describe and understand the interplay between managerial hybrid levels within a decentralized organization structured in CDs grouped in divisions, (2) inform health care systems and managers that are considering implementing clinical managerial levels, and (3) contribute to theory by discussing these findings in relation to hybrid management, clinical directorates, and decentralization.

### Theoretical background

Clinical directorate (CD) were introduced to involve health professionals in management [2–4, 10], originating from functional specialty units for cost-control at Johns Hopkins, USA, in 1973 [20]. Since then, the model has spread globally with contextual variations [13] as a shift from “professional bureaucracy to a divisionalized form” [21]. CDs led by hybrid managers are intended to improve resource allocation [22] and efficiency [23].

Hybrid managers combine professional and managerial logics [6], balancing the autonomy of highly trained professionals [24–26], who historically resisted changes threatening autonomy [21, 27–29]. With clinical and managerial expertise [30] and a patient focus [31], hybrid managers are expected to bridge the divide between managerial and medical professional logics [32, 33].

**Fig. 1:**
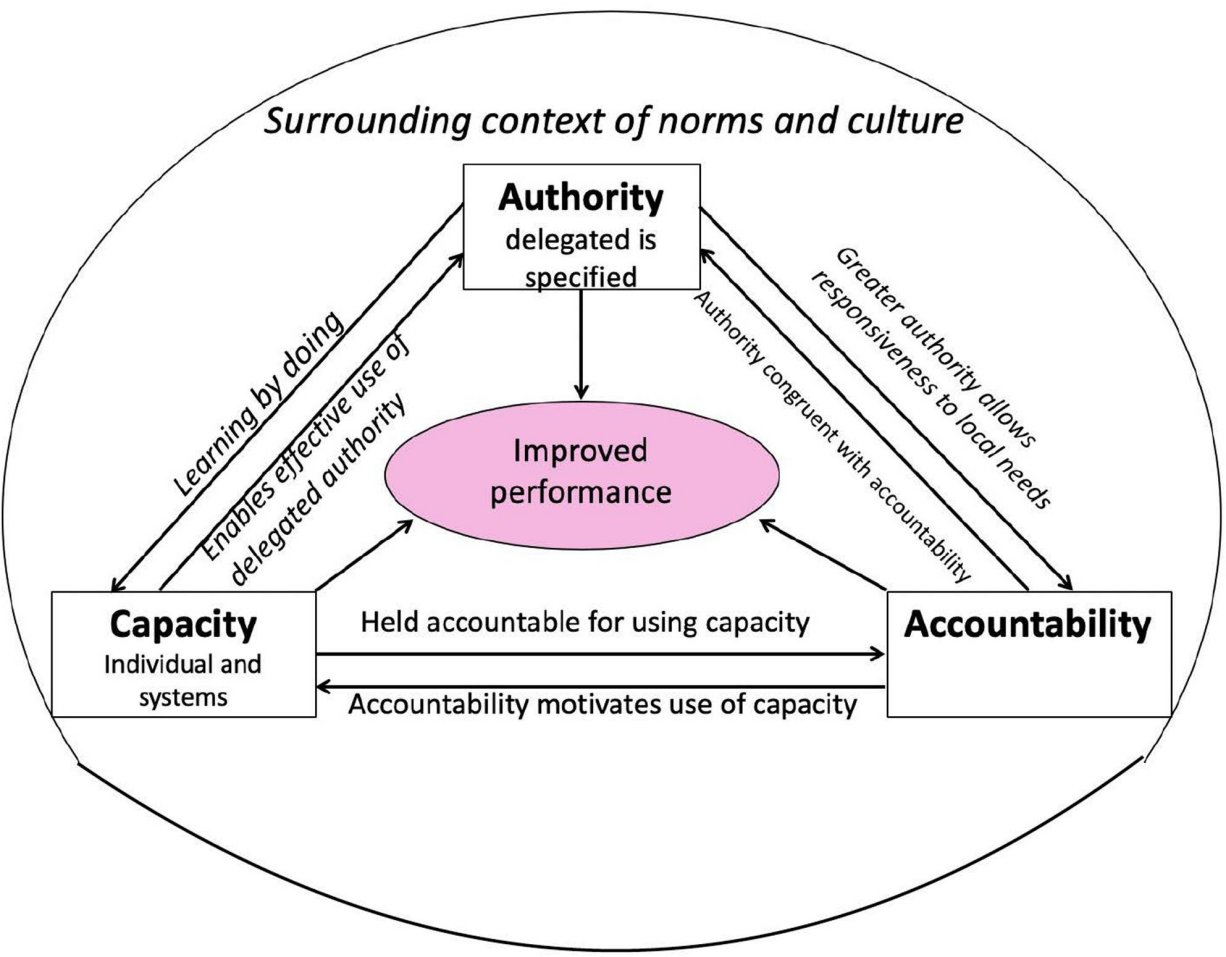
The interaction between delegated authority, accountability, and capacity (both individual and organisational) in CDs [37]

Decentralizing decision-making is assumed to enhance responsiveness to local needs [34, 35]. CDs with hybrid managers given a delegated authority should strengthen such decentralization [36]. A scoping review [37] adapted Bossert’s decision space model [38] to illustrate how delegated authority interacts with accountability and managerial capacity at individual and organizational levels (Fig. 1).

## Methods

### Study design

A sequential explanatory mixed-methods design with both quantitative and qualitative analyses of two surveys was applied to examine how clinical directors perceived the introduction of a new divisional hybrid-managerial level within a decentralized healthcare organization. The quantitative survey data were first analyzed to identify overall patterns and differences over time, followed by a directed qualitative content analysis of open-text responses to explain and elaborate on the quantitative results. This design allowed the quantitative findings to guide the qualitative interpretation, thereby enhancing the validity of conclusions drawn from both strands, and enabled comparisons over time [39, 40].

### Survey instrument

An ad hoc questionnaire [41, 42] was specifically developed to evaluate the effects of the reorganization in a structured, three-step process: (1) the three main items were derived directly from the reform’s stated objectives - (a) freeing time for patient care and development, (b) reducing administrative burden, and (c) strengthening central administrative support; (2) the draft questionnaire was reviewed and refined during a workshop with the senior management team to ensure face validity and relevance; and (3) a panel of experienced clinical directors tested the instrument for clarity, feasibility, reliability, and content validity [43].

Each of the three items was formulated as a statement rated on a five-point Likert scale [44] ranging from “strongly disagree” (1) to “strongly agree” (5), with an additional “Don’t know / not applicable” option. Immediately following each item, respondents could elaborate through an optional open-text box, allowing for qualitative input linked to the same topic. Although internal consistency (Cronbach’s α) was considered, reliability testing was not statistically performed due to the limited number of single-item constructs; instead, reliability was addressed through pretesting and content validation.

### Participants

All ninety-five (20% male, 80% female) clinical directors of the organization were invited to participate. This census approach ensured representation across all divisions—primary healthcare, mental healthcare, geriatrics and somatic care, habilitation and assistive technology, and research and development. Respondents shared equivalent managerial responsibilities regardless of clinical background or unit size, providing a comprehensive view of perceptions across contexts. They all, to various extents, worked as clinicians with detailed understanding of daily operations and the issues involved for all clinical and administrative staff. At the time of the first survey, research centers were separate entities in each clinical division but a research and development division was under formation.

### Data collection

Data were collected twice through anonymous web-based surveys (Webropol): in May 2018 and November 2019. Each survey remained open for ten days, with a reminder sent after five. Participation was voluntary, and no identifying information was stored. The questionnaire is available in Additional File 1.

### Data analysis

Quantitative analysis included descriptive statistics and non-parametric tests—Mann-Whitney exact tests and Fisher’s exact tests—to compare groups and results between 2018 and 2019 across divisions. To describe the distribution and uncertainty of responses, three indices were calculated: (1) Polarity Index (PI): balance between positive and negative responses; (2) Intensity Index (II): strength of agreement or disagreement; and (3) Uncertainty Index (UI): proportion of neutral or “Don’t know” responses. These indices provide additional insight into response patterns to better understand the distribution of responses and uncertainty in the data, particularly in the presence of small samples or uneven distributions [4, 45].

All analyses were based on available data; missing values and “Don’t know” responses were excluded from inferential tests but included when computing the UI to reflect uncertainty [4, 45]. Open-ended responses were analyzed using directed content analysis [46, 47], guided by the three reform objectives. Text segments were coded as advantages or disadvantages relative to each objective. Integration of quantitative and qualitative results followed a sequential explanatory strategy: qualitative findings were used to interpret and refine quantitative trends, ensuring a coherent understanding of the perceived effects of the organizational change.

### Ethical considerations

The study was conducted in accordance with the ethical principles of the Declaration of Helsinki (World Medical Association, 2013). The study was performed as a part of a larger project on management decentralization, the research plan of which has been evaluated and endorsed by the Stockholm Regional Research Ethics Board (DNr 2018/98-31/5) which is a part of The Swedish Ethical Review Authority”.

The surveys were performed as a part of ordinary operations. All methods were performed in accordance with relevant guidelines and regulations, including informed consent to participate in the study from all the participants.

## Results

### Sample characteristics

Of the 95 clinical directors invited, 80 (84%) completed the first survey in May 2018 and 92 (97%) completed the second in November 2019. Approximately two thirds of the respondents were women. Clinical directors in primary healthcare (PHC) represented the largest group in both surveys (74% and 69%). Their units were typically smaller, while those in mental healthcare services (MHCS) and habilitation and assistive technology (HA) managed larger teams with a wider span of control. Almost half of the respondents in 2018 (46%) and two thirds in 2019 (65%) had less than two years of experience in their role, Table 2.

**Table 2:** Characteristics of the study samples, surveys 1 and 2

Characteristics Response rate (*n* of total 95 in %)	Survey 1 May 2018 *n* = 80 (84%)	Survey 2 November 2019 *n* = 92 (97%)
Divisional affiliation		
Primary healthcare (PHC)	59 (74%)	63 (69%)
Mental healthcare services (MHCS)	10 (12%)	10 (11%)
Geriatric and somatic care (GASE)	7 (9%)	9 (10%)
Habilitation and Assistive technology (HA)	4 (5%)	5 (5%)
Research and Development (RD)^*^	Under formation	5 (5%)
Experience as clinical director		
≤2 years	37 (46%)	60 (65%)
3–10 years	27 (34%)	24 (26%)
11–15 years	9 (11%)	1 (1%)
≥16 years	7 (9%)	6 (8%)
Span of control		
<20 employees	4 (5%)	8 (9%)
21–50 employees	37 (46%)	35 (38%)
51–200 employees	28 (35%)	34 (38%)
>201 employees	11 (14%)	14 (15%)
Subordinate managers in CDs		
None	17 (21%)	19 (21%)
Yes	63 (79%)	73 (79%)
Range [1–35]		
Median PHC/GASE/RD 6; MHCS/HA 27		

PHC = Primary healthcare; MHCS = Mental healthcare services; GASE = Geriatrics, Advanced palliative home care, Somatic specialist care, Emergency centers; HA = Habilitation and assistive technology; RD = Research and development; *) from 1 Oct 2017

### Objective 1: Freeing up time for patient care and development

In 2018, 72% of respondents disagreed or strongly disagreed that the new organization freed up more time for patient care and development, while 6% agreed. In 2019, disagreement decreased to 47%, and agreement increased to 24%. The proportion of “Don’t know” responses rose slightly from 22% to 28%. All the data are presented in Additional File 2.

Open-text responses (*n* = 52) reflected both positive and negative views. Some clinical directors reported improved communication and collaboration within divisions, while others mentioned an increased number of meetings and persistent time constraints at the clinical level.

### Objective 2: Reduced administrative burden

In 2018, 74% disagreed or strongly disagreed that the administrative burden had decreased, compared with 57% in 2019. Agreement increased from 10% to 15%. “Don’t know” responses increased from 15% to 24%.

Comments (*n* = 58) indicated that certain coordination processes had improved, especially related to joint plans and management routines, but administrative demands remained high. Respondents frequently mentioned unclear responsibilities and lack of digital support as contributing factors.

### Objective 3: Strengthening central administrative support

In 2018, 49% disagreed or strongly disagreed that central support had improved; this decreased to 37% in 2019. Agreement increased from 33% to 42%. Uncertainty (“Don’t know”) rose from 18% to 22%.

Among 63 comments, respondents noted better access to HR and patient safety expertise in some divisions but described the overall support as inconsistent. Several emphasized that outcomes depended on individual contacts rather than standardized systems.

### Comparison across divisions

Divisional differences were evident. PHC respondents expressed the highest dissatisfaction with administrative workload, while MHCS directors more frequently mentioned improved coordination. GASE and HA respondents described mixed experiences—some progress in HR and finance continuity but ongoing coordination challenges. The RD division, established in 2017, reported improved strategic focus by 2019.

### Comparison over time (May 2018-November 2019)

Across all divisions, positive responses increased modestly between 2018 and 2019. Administrative burden and uncertainty decreased slightly but remained notable. MHCS showed the largest relative improvement in perceptions of leadership and support, whereas PHC continued to report administrative overload.

### Statistical analysis

Non-parametric tests (Mann–Whitney exact and Fisher’s exact) indicated significant changes between 2018 and 2019 for Objective 1 (*p* < 0.001) and Objective 2 (*p* = 0.0046) at the total level. No statistically significant change was found for Objective 3. Division-specific results showed that PHC and MHCS experienced significant variation in perceptions of time for patient care (Objective 1). Changes in other divisions were non-significant. All the statistical comparisons between 2018 and 2019 and their corresponding significance levels are summarized in Table 3.

**Table 3:** Significance levels for the three objectives total and per division

	Objective 1: Free up time		Objective 2: Reduce admin		Objective 3: Central support	
	M-W *p* value	Chi2 *p* value	M-W *p* value	Chi2 *p* value	M-W *p* value	Chi2 *p* value
Total	0.0001	0.0001	0.0046	0.056	0.1348	0.4766
PHC	0.0003	0.0009	0.0311	0.1771	0.0203	0.1708
MHCS	0.0105	0.0555	0.3276	0.809	0.5382	0.7613
GASE	0.7336	0.8238	0.7308	1	0.4261	0.5668
HA	1	1	0.3016	0.1905	1	0.5714

The Polarity (PI), Intensity (II), and Uncertainty (UI) indices are summarized in Table 4. Between 2018 and 2019, PI generally increased, indicating more balanced distributions of responses, while II decreased, suggesting less polarization. UI rose in several divisions, reflecting growing uncertainty about the impact of the new organization.

**Table 4:** Polarity (PI), intensity (II) and uncertainty (UI) indices

Division	PI_2018	PI_2019	II_2018	II_2019	UI_2018	UI_2019
Total	*0.08/0.67/0.32	0.53/0.88/0.54	43/23/44	15/16/21	22/18/4	28/21/14
PHC	0.12/0.53/0.4	0.56/0.7/0.6	48/21/37	18/16/18	21/19/5	25/22/15
MHCS	0/0.5/0	0.75/0.6/0.11	40/30/80	10/10/20	10/10/0	30/20/0
GASE	0/1/0.39	0/0.16/0.77	29/14/57	22/11/34	14/14/0	33/22/22
HA	0/0.5/0	0.33/0/0.5	0/50/50	0/0/20	50/25/0	20/40/40
RD	-	1/0.67/0	-	0/40/20	-	60/0/0

*) Indices in frames corresponding to objective 1/objective 2/objective 3

## Discussion

This study explored how clinical directors perceived the introduction of a new divisional hybrid-managerial level in a large, decentralized public healthcare organization. The results show that while the reform improved coordination and access to specialized expertise, it did not substantially reduce administrative workload or free time for patient care. The findings illuminate the complex interplay between managerial and professional logics in multi-level hybrid management structures.

### Freeing up time for patient care and development

The findings indicate that the introduction of the divisional hybrid-managerial level did not substantially free up time for patient care or organizational development, particularly in PHC and MHCS. This aligns with earlier research showing that hybrid managers often struggle to balance clinical and managerial demands and may encounter tensions related to professional autonomy and role ambiguity [1, 2, 13, 21]. Several clinical directors reported that increased meetings and structural complexity added to the administrative workload rather than reducing it, consistent with observations in decentralized hybrid-management settings [8, 22]. However, improvements in collaborative work were noted in MHCS, where hybrid managers with clinical backgrounds facilitated joint quality and process improvement, supporting evidence on the importance of effectively enabling hybridity in core processes [48].

The results further reflect well-established challenges in decentralized governance. While decentralization can enhance responsiveness and local decision-making [49, 50], it requires clearly defined responsibilities, delegated authority and sufficient capacity at all levels [37, 38]. In this study, ambiguity regarding roles and authority—particularly in administrative areas—limited the intended benefits, echoing previous findings that unclear decentralized structures can create confusion and undermine decision-making [51, 52]. Survey findings from 2019 confirmed this, with many clinical directors reporting uncertainty about their authority following the introduction of the divisional level. Nonetheless, several PHC and MHCS directors highlighted those joint processes benefitted from the clinical competence of divisional hybrid managers, in line with Prenestini et al. [4].

Matching delegated authority with adequate local support is also critical [38, 53]. Yet, increased spans of control and insufficient support in PHC led to greater administrative burden, consistent with Wong’s argument that wide control spans can overwhelm managers in complex healthcare environments [54].

Overall, the results suggest that decentralization may support improved management structures only when authority, responsibilities and capacity are clearly communicated and supported. In this case, blurred boundaries between managerial levels and unclear delegation limited intended efficiency gains, reinforcing the importance of clarity and structured support in decentralized hybrid-managerial systems [50, 51].

### Reduction in administrative burden

The study found only limited improvements in reducing administrative burden, with persistent challenges across divisions. Although some PHC respondents reported modest gains in coordination and communication, administrative tasks remained substantial. This aligns with Exworthy et al. [35], who observed that decentralization and the introduction of hybrid managerial roles rarely produce the intended reductions in administrative workload. The addition of a new managerial tier may instead increase bureaucratic complexity, as shifts in power and coordination requirements generate new administrative demands [3, 6].

Clinical directors’ reports of increased bureaucracy mirror earlier findings that hybrid models may unintentionally amplify administrative responsibilities due to complex governance arrangements [1]. While shared initiatives, particularly around patient safety plans, improved in certain divisions, these gains were unevenly distributed. Such variation may reflect heterogeneity between divisions, suggesting that hybrid managers face challenges in standardizing processes across diverse clinical settings — a pattern also noted by Braithwaite and Westbrook [1].

### Strengthening of central administrative support

Changes in central administrative support between 2018 and 2019 were limited. Although respondents reported improvements in selected areas, notably HR and patient safety, support was inconsistent and often dependent on individual staff members rather than systematic organizational strengthening. This finding is consistent with previous research showing that hybrid managers frequently struggle to secure coherent and reliable administrative support across all domains [30–32].

Some improvements align with Montgomery’s argument that well-implemented hybrid structures can enhance administrative services [16]. However, broader organizational cultures that resist change — as described by Christensen and Laegreid [8] — may weaken such benefits. In this study, the introduction of a divisional hybrid-managerial layer and the resulting power shifts contributed to uncertainty in support structures and impeded the intended strengthening of central functions.

### Organizational culture and resistance to change

Across divisions, resistance to structural change emerged as a prominent theme, characterized by unclear role boundaries and professional concerns regarding managerial oversight. This mirrors long-standing observations of professional resistance within healthcare organizations [21, 24, 25, 27]. The ambiguity generated by the new divisional level heightened tensions between clinical and managerial expectations. Such tensions are well captured by Packwood et al. [22] and Olakivi and Niska [33], who describe competing professional and managerial logics that may undermine hybrid role functioning.

Despite perceived improvements in leadership and coordination in MHCS and PHC, role ambiguity persisted, particularly regarding administrative responsibilities. These tensions reflect the difficulty hybrid managers face when navigating overlapping clinical and managerial identities. The challenge becomes amplified when hybrid managers share the same professional background as those they supervise, as occurred at both the divisional and CD levels in this study. This structural proximity may obscure authority lines and intensify role conflict.

The findings underscore the need to identify enablers that strengthen hybrid capacity—such as clearer role delineation, training, and organizational support—consistent with Sartirana and Giacomelli’s review [48] and studies highlighting the importance of hybrid-manager training in decision-making [10].

### Implications for healthcare management

The results suggest several implications for healthcare organizations considering divisional structures and hybrid managerial roles. First, while hybrid management can enhance coordination and leadership at higher organizational levels, it does not inherently reduce administrative burden or free time for patient care at the CD level. Structural reforms must therefore account for potential unintended consequences, including increased complexity and workload.

Second, consistent with Packwood et al. [22], hybrid roles require well-defined responsibilities and strong support systems, particularly in decentralized contexts where authority and accountability may otherwise become diffuse. Training and capacity-building—highlighted in prior research [10]—are essential to ensure that hybrid managers can navigate their dual responsibilities effectively.

Third, successful hybrid management depends on an organizational culture that values both professional and managerial contributions. As Reay and Hinings [29] emphasize, fostering alignment between these logics is critical for hybrid-role effectiveness. Ensuring that hybrid managers receive the tools and support they need is central to this alignment.

Finally, echoing the work of Correia and Denis [13], future research should examine the micro-level interactions and processes that shape hybrid managers’ day-to-day work, including how they negotiate authority, influence peers, and navigate ambiguous structures.

### Limitations and challenges

This study has several limitations. The surveys were conducted at only two time points (2018 and 2019), limiting the ability to assess long-term effects of the reorganization. Furthermore, the study was conducted within a single regional healthcare provider, which may limit generalizability to other settings. Broader, multi-site studies with longer follow-up periods would provide more comprehensive insights.

The use of self-reported data introduces the potential for response bias, although triangulation with qualitative comments strengthens interpretive validity. The analytical approach—using the Mann–Whitney exact test, Fisher’s exact test, and polarity, intensity and uncertainty indices—was well suited to small-sample ordinal data but carries methodological limitations. Mann–Whitney and Fisher’s tests are robust for small samples but may lack power to detect subtle differences, while the interpretation of the indices requires contextual judgement. Future studies with larger sample sizes could increase statistical power and enhance the reliability of distributional comparisons.

## Conclusion

This study examined how clinical directors perceived the introduction of a divisional hybrid-managerial level in a large, decentralized healthcare organization. The results show that while the reform improved coordination and leadership capacity, it also introduced new layers of complexity and uncertainty.

Effective hybrid management requires more than professional alignment — it depends on clear role definitions, balanced delegation of authority, and consistent organizational support. Without these conditions, hybrid structures risk increasing administrative burden rather than achieving the intended efficiency gains.

This study makes a theoretical contribution primarily to the literature on hybrid management and decentralized healthcare governance. While previous research has largely focused on the emergence, identity, and performance of individual hybrid roles, this study extends theory by examining the interplay between multiple hybrid-managerial levels within the same organization. By empirically demonstrating how the introduction of a divisional hybrid-managerial layer reshapes authority, accountability, and role clarity at the clinical directorate level, the findings contribute to a more nuanced understanding of multi-level hybridity in complex professional bureaucracies.

The study further advances decentralization theory by showing that the effectiveness of delegated authority depends not only on professional alignment but also on the configuration of hybrid roles across organizational levels. The results illustrate how layering hybrid roles without clearly defined boundaries and support mechanisms may generate ambiguity and administrative burden, thereby constraining the intended benefits of decentralization. In this way, the study refines existing frameworks that link decision space, accountability, and managerial capacity by highlighting the importance of vertical role coherence in hybrid governance structures.

By integrating insights from hybrid management theory and decentralization research, this study contributes to theory development on how professional and managerial logics are negotiated across hierarchical levels, rather than within isolated roles. It thus responds to calls for more fine-grained, process-oriented analyses of hybrid management in healthcare organizations and offers a conceptual basis for understanding when and why hybrid governance arrangements enable—or hinder—organizational effectiveness. For healthcare managers and policymakers, the study underscores the importance of designing hybrid roles that combine professional credibility with managerial capability, supported by coherent governance and communication systems.

## Supplementary Information

Below is the link to the electronic supplementary material.

Supplementary Material 1

Supplementary Material 2

## Data Availability

The datasets used and/or analyzed during the current study, data associated, and surveys are available from the corresponding author upon reasonable request.

## Abbreviations

CD: Clinical Directorate
CEO: Chief Executive Officer
NPM: New Public Management
HR: Human Resources
PHC: Primary healthcare
MHCS: Mental healthcare services
GASE: Geriatrics, Advanced Palliative Home Care, Somatic Specialist Care, Emergency centers
HA: Habilitation and Assistive technology
RD: Research and Development
M-W: Mann‒Whitney Exact test
PI: Polarity Index
II: Intensity Index
UI: Uncertainty Index
