# Supplementary material for: Challenges in hybrid management in healthcare: a study of the interplay between divisional managers and clinical directors in a decentralized healthcare organization in Sweden
Source: BMC Health Serv Res. 2026 Jan 12;26:145. doi: 10.1186/s12913-025-13977-y (PMC12849398; doi:10.1186/s12913-025-13977-y)
Supplement: Supplementary file 1 — Supplementary Material 1 [file 12913_2025_13977_MOESM1_ESM.docx]

Additional File 1

Translation of Survey Instrument

**1. I belong to the following operational area (Division):**
☐ Primary Care
☐ Psychiatry
☐ Habilitation and Assistive Technology
☐ Somatic Specialist Care

**2. I have been clinical director for:**
☐ 0–2 years (appointed after the new VO-structure)
☐ 3–10 years
☐ 10–15 years
☐ More than 15 years

**3. Number of employees at my unit (CD):**
☐ Fewer than 20
☐ 16–50
☐ 51–200
☐ More than 201

**4. Are there unit managers at my CD?**
☐ No
☐ Yes, number: ______________________________

**5. Please indicate the statement that best matches your opinion:**
*The new divisional organization has freed up time for care and organizational development at the CD level.*
☐ Don’t know
☐ Strongly disagree
☐ Somewhat disagree
☐ Somewhat agree
☐ Strongly agree

**Comments:**

**6. Please indicate the statement that best matches your opinion:**
*The new divisional organization has improved coordination and reduced administrative workload at the CD level.*
☐ Don’t know
☐ Strongly disagree
☐ Somewhat disagree
☐ Somewhat agree
☐ Strongly agree

**Comments:**

**7. Please indicate the statement that best matches your opinion:**
*The new divisional organization has strengthened the shared operational central support (competence and responsiveness in various matters).*
☐ Don’t know
☐ Strongly disagree
☐ Somewhat disagree
☐ Somewhat agree
☐ Strongly agree

**Comments:**
