# Supplementary material for: Challenges in hybrid management in healthcare: a study of the interplay between divisional managers and clinical directors in a decentralized healthcare organization in Sweden
Source: BMC Health Serv Res. 2026 Jan 12;26:145. doi: 10.1186/s12913-025-13977-y (PMC12849398; doi:10.1186/s12913-025-13977-y)
Supplement: Supplementary file 2 — Supplementary Material 2 [file 12913_2025_13977_MOESM2_ESM.docx]

Additional File 2

Clinical directors’ responses to questionnaire May 2018 and November 2019

Responses % PHC MHCS GASE HA RD^*^ TOTAL

1. The new divisional level has freed up time for healthcare and development at the clinical level

May 2018

SA 0 0 0 0 - 0

A 8 0 0 0 - 6

D 20 50 57 50 - 29

SD 48 40 29 0 - 43

N 24 10 14 50 - 22

November 2019

SA 2 0 0 0 0 1

A 25 40 0 20 20 24

D 32 20 45 60 20 33

SD 16 10 22 0 0 14

N 25 30 33 20 60 28

2. The new divisional level has made the coordination reduce the administration at the clinical level

May 2018

SA 0 0 0 0 - 0

A 12 0 0 25 - 10

D 26 40 29 75 - 30

SD 43 60 57 0 - 44

N 19 0 14 0 - 16

November 2019

SA 2 0 0 0 20 2

A 16 10 0 25 40 15

D 30 40 33 25 40 32

SD 24 40 45 0 0 25

N 28 10 22 50 0 26

3. The new divisional level has strengthened joint administrative support (competence and speed in getting help in various administrative matters) at clinical level

May 2018

SA 4 30 0 25 - 8

A 24 30 43 0 - 25

D 36 30 29 25 - 34

SD 17 0 14 25 - 15

N 19 10 14 25 - 18

November 2019

SA 5 10 0 0 40 7

A 41 40 11 0 20 35

D 21 30 56 60 40 28

SD 11 0 11 0 0 9

N 22 20 22 40 0 21

PHC=Primary healthcare; MHCS=Mental Healthcare Services; GASE=Geriatrics, Advanced Palliative Home Care, Somatic specialist care, and Emergency centers; HA=Habitation and Assistive Technology; RD=Research and Development; SA=Strongly Agree; A=Somewhat Agree, D=Somewhat Disagree; SD=Strongly Disagree; N=Don’t know; *) established 1 Oct 2017, under formation at the time for the first survey in May 2018
